# Supplementary material for: Administration of Nicotinamide Mononucleotide (NMN) Reduces Metabolic Impairment in Male Mouse Offspring from Obese Mothers
Source: Cells. 2020 Mar 25;9(4):791. doi: 10.3390/cells9040791 (PMC7226525; doi:10.3390/cells9040791)
Supplement: Supplementary file 1 [file cells-09-00791-s001.pdf]

Administration of nicotinamide mononucleotide (NMN) reduces metabolic impairment in male mouse offspring from obese mothers

Golam Mezbah Uddin[1], Neil A.Youngson[1], Sabiha S. Chowdhury[1], Christopher Hagan[1] David A. Sinclair[1, 2], Margaret J. Morris[1*]

[1]Department of Pharmacology, School of Medical Sciences, UNSW Sydney, Australia
[2]Department of Genetics, Paul F. Glenn Laboratories for the Biological Mechanisms of Aging, Harvard Medical School, Boston, MA, United States

Supplementation material

Table S1: Primer sequences of genes examined

Gene	Forward	Reverse
Sirt1	TGT AAG TTA CTG CAG GAG TGT AAA	GCA TAG ATA CCG TCT CTT GAT CTG AA
Pgc1a	TATGGAGTGACATAGAGTGTGCT	CCACTTCAATCCACCCAGAAAG
Pparg	TCGCTGATGCACTGCCTATG	GAGAGGTCCACAGAGCTGATT
Fasn	AAGCCGTTGGGAGTGAAAGT	CAATCTGGATGGCAGTGAGG
Cd36	TGG CCT TAC TTG GGA TTG G	CCA GTG TAT ATG TAG GCT CAT CCA
Mpc1	GTG CTG CAG CCT TTC TCA C	GTT CCC ACA AAG GCA TCA C
Acc1	GCC TCC GTC AGC TCA GAT AC	ATT CTG GCT GGA GAA GCC ACA
Acc2	CAT ACA CAG AGC TGG TGT TGG ACT	CAC CAT GCC CAC CTC GTT AC
Cpt1a	CAG AGG ATG GAC ACT GTA AAG G	CGG CAC TTC TTG ATC AAG CC
Hadh	TTG CGC TCC ATG TCC TCC TC	GAC TCT CCT CAA TTC CCT TC
Gapdh	AGGTCGGTGTGAACGGATTTG	TGTAGACCATGTAGTTGAGGT
Ywhaz	GAAAATGAAGGGTGACTACTAC	CTGATTTCAAATGCTTCTTG
